# A simple approach to thumb amputation reconstruction at metacarpal base with toe transfer, two case reports

**DOI:** 10.1016/j.ijscr.2020.02.037

**Published:** 2020-02-21

**Authors:** Viet Tan Nguyen, Van Doan Le, Viet Tien Nguyen

**Affiliations:** Upper Extremity Trauma and Microsurgery Department, 108 Military Central Hospital, 1 Tran Hung Dao Street, Hanoi, Viet Nam

**Keywords:** Toe transfer, Thumb amputation, Thumb reconstruction

## Abstract

**Introduction:**

Reconstruction for thumb amputation at the metacarpal base by toe transfer is challenging. To restore a thumb with normal or near-normal length, the reconstruction plan usually involves a complicated and challenging process either in two stages (stage 1: resolving soft tissue and bone defect; stage 2: toe transfer) or a single stage by using two free flaps (one free soft tissue flap and one toe flap). However, is it necessary to restore the full length of the thumb for functional and aesthetic achievement?

**Presentation of cases:**

Two male patients (21 and 22 years old) had a thumb amputation at the metacarpal base. We accepted the shortened metacarpal length and performed reconstruction in one stage by trimmed great toe flap, at the level of the metatarsophalangeal joint. The first phalanx of toe flap was fused with the first metacarpal base. On long-term follow-up, both patients were able to return to daily activities, work and had a good cosmesis.

**Discussion:**

With our reconstruction technique, two reconstructed thumbs were functionally similar to a thumb amputation group 1 of Campbell-Reid. Using trimmed great toe flap, the tip of our reconstructed thumbs looks like that of a normal thumb. Both patients were satisfied.

**Conclusion:**

Accepting length shortening, the reconstruction for thumb amputation at the metacarpal base by toe transfer could be done more easily and simply in a single stage.

## Highlights

• Reconstruction for thumb amputation at the metacarpal base by toe transfer is challenging.
• To restore a thumb with normal or near-normal length, the reconstruction plan usually involves a complicated and challenging process either in two stages (stage 1: resolving soft tissue and bone defect; stage 2: toe transfer) or a single stage by using two free flaps (one free soft tissue flap and one toe flap).
• We accepted the shortened metacarpal length and performed reconstruction in a single stage by trimmed great toe flap, at the level of the metatarsophalangeal joint. The first phalanx of toe flap was fused with the first metacarpal base.
• The reconstructed thumbs were functional similarly to a thumb amputation group 1 of Campbell-Reid. The tip of the reconstructed thumb looks like that of a normal thumb.
• With this technique, the reconstruction process could be done more easily and simply in a single stage.

The following case report has been reported in line with the SCARE criteria [1].

## 1 Introduction

The thumb constitutes approximately 40–50% of the function of the hand. Therefore, traumatic loss of the thumb causes significant function disability and aesthetic deficits, especially if it is at the base of the metacarpus. Reconstruction for thumb amputation at the metacarpal (MC) base is extremely challenging. Pollicization of the index finger is usually the recommended technique for the reconstruction for the thumb at this level [[2], [3], [4]]. However, pollicization is impossible in some instances including an injured superficial palmar arch, amputation of other fingers and the aesthetic requirement. For these instances, toe transfer is probably a reasonable option. The main difficulties of thumb reconstruction at MC base are length and soft tissue coverage. To restore the thumb with length similar to one on the normal hand, some authors suggested a reconstruction process involving either two stages or a single stage by applying two free flaps (1 free soft tissue flap and 1 toe flap) at the same time [[5], [6], [7], [8], [9]]. These methods might be complicated for patients in developing countries. To reconstruct the thumb amputation at the MC base in one stage and minimize the foot morbidity, we decided to harvest the trimmed great toe (TGT) flap at metatarsophalangeal (MTP) joint and fuse the first phalanx of toe flap with the first MC base. In this article, we present the surprising results of our two cases.

## 2 Presentation of case

### 2.1 Case 1 (Fig. 1A–I)

A 21-year-old male was hospitalized to our hospital in January 2011. He had an amputation at the first MC base, a defect of second MC bone, and a 4 cm × 5 cm bad scar at the posterior-lateral side of the right hand due to labor accident two months before admission. All the right thenar muscles were lost.

**Fig. 1:**
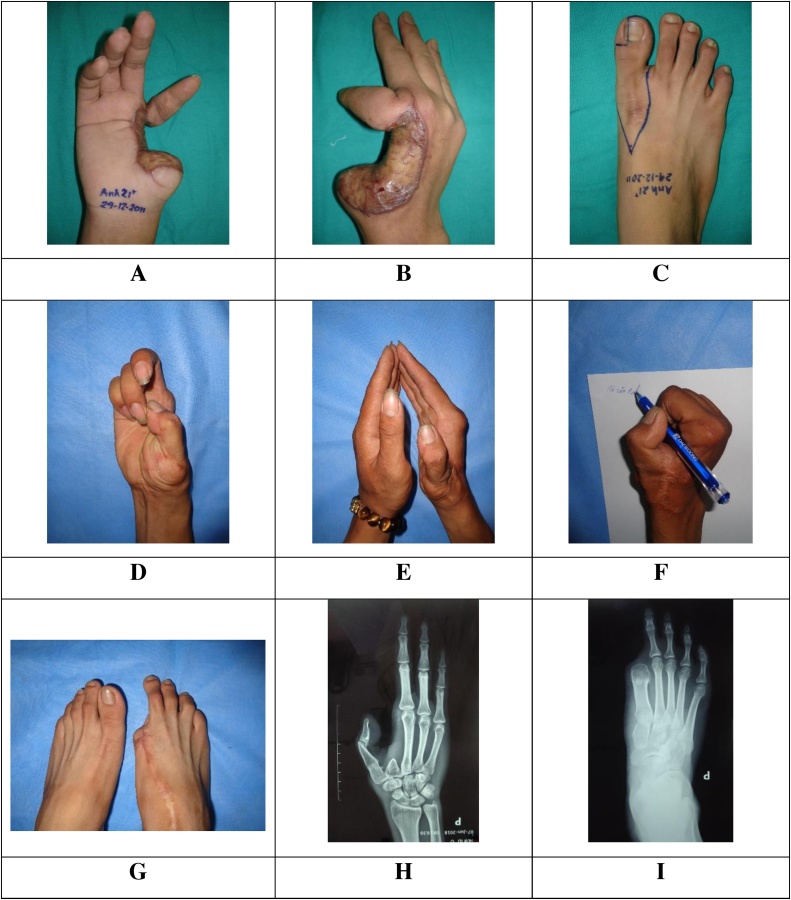
Pre-operative and post-operative images of patient 1. A-C: pre-operative images. D-I: post-operative images.

Surgical technique: The right thumb was reconstructed by free right TGT flap. The first phalanx of the TGT flap was fused with the first MC base by 2 K-wires. The scar was excised and covered by a fillet flap from the index finger. The 2 K-wires was removed 10 weeks following the first operation.

### 2.2 Case 2 (Fig. 2A–I)

A 22-year-old male lost his right thumb at the MC base due to a press machine accident. He was referred to our hospital in December 2011.

**Fig. 2:**
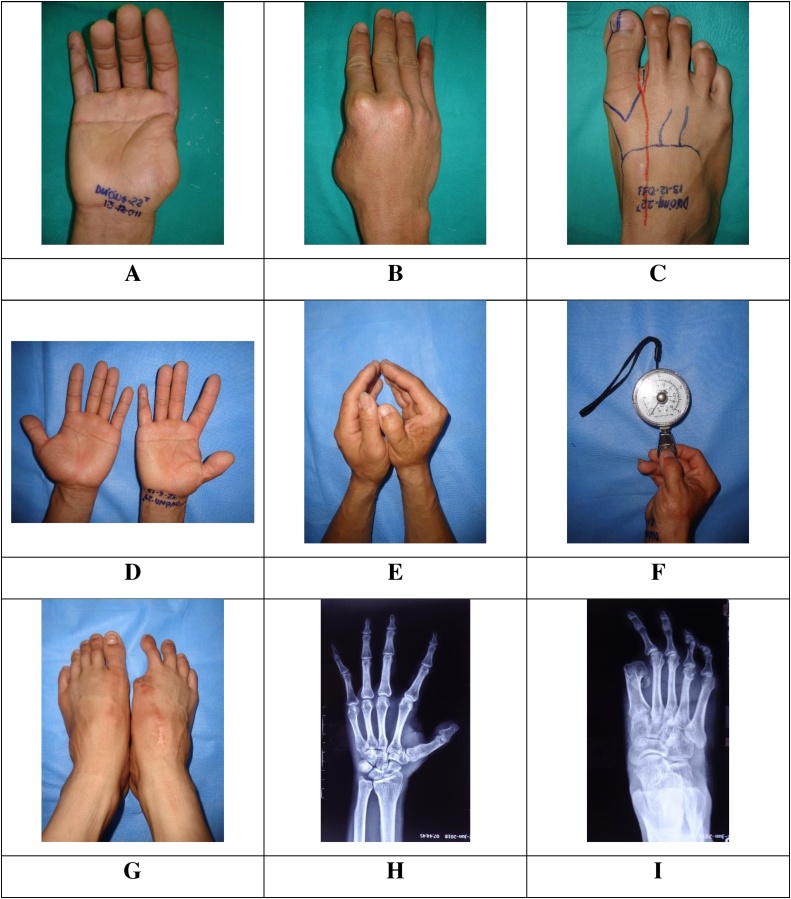
Pre-operative and post-operative images of patient 2. A-C: pre-operative images. D-I: post-operative images.

Surgical technique: For thumb reconstruction, a free right TGT flap was harvested and transferred to first MC base by microsurgical techniques. The first phalanx of toe flap was fused with the first MC base by 2 K-wires that would be removed 10 weeks following the first operation.

Long-term follow-up: Both toe transfers survived. After 7 years follow-up, we re-evaluated the functional outcomes of the reconstructed thumbs and foot morbidities (Table 1). Both patients returned to the job they had worked before the accident. The foot morbidity was trivial.

**Table 1:** Long-term outcomes.

Outcomes	Patient 1	Patient 2
Range of motion of interphalangeal joint of the neothumb	25°	40°
Pinch power (% of uninjuried side)	61%	57%
Grip power (% of uninjuried side)	86%	76%
Kapandji opponent score	5	8
Static 2 point discrimination	7 mm	14 mm
Shorter length of the neothumb with the one of contralateral side	4 cm shorter	3 cm shorter
Michigan hand outcomes questionnaire score: - overall hand function	75	50
- activities of daily living	91	85
- work performance	100	60
- pain	0	10
- aesthetics	88	63
- satisfaction	100	50
- overall score	92	66
The foot and ankle disability index score	0/100	17/100

## 3 Discussion

In our report, two patients suffered from crush injuries due to labor accident. The thenar muscles were destroyed. With pollicization, motion and sensory of the reconstructed thumbs can be quickly restored [3]. However, pollicization is contraindicated in instances that the palmar artery arch is injured or the other fingers are lost. In the aesthetic aspect, pollicization also does not provide a normal appearance because the hand still lacks fingers. Therefore, in such instances, toe transfer is an option.

In medical literature, many authors mentioned toe-to-thumb transfer to restore thumb amputation at the metacarpophalangeal (MCP) joint level but reports on thumb reconstruction at the MC base by toe transfer is limited and have small sample sizes. To restore the thumb with normal or near-normal length, the toe transfer procedure is very challenging because of bone and tissue defect. Hence, some authors decided to reconstruct in two stages or apply two free flaps (a toe flap and a tissue flap) in a single stage. Sabapathy reconstructed the thumb amputation at the carpometacarpal joint level by groin flap in the primary stage and trans-metatarsal second toe transfer in the secondary stage [5]. Woo reconstructed by trans-metatarsal whole great-toe transfer or trans-metatarsal second toe transfer after iliac bone graft and groin flap [8]. The disadvantage of these above methods is long reconstructive duration. Moreover, the trans-metatarsal great toe flap will leave a significant morbidity to foot due to destruction of windlass mechanism. Oña et al. used two free flaps (a toe flap and a soft tissue flap) for thumb reconstruction at the level of the MC base on two patients [7]. Disadvantages of Oña’s technique are the need for two sets of recipient vessel, longer operative time, and a more challenging technique.

In this report, our two patients are manual workers with limited income. To help reduce reconstructive duration, cost, and foot morbidity, we chose to reconstruct the thumb with a shorter length by TGT flap simply in one stage.

In reality, a thumb amputation distal to MCP joint, leaving at least proximal half of proximal phalanx (amputation group 1 according Campbell-Reid classification) does not need to be reconstructed and still can provide and support enough function to hand [10]. Based on this rationale, the two reconstructed thumbs in our report have the length like a thumb amputation group 1 of Campbell-Reid. Hence, they could absolutely provide the function to the hand.

To achieve the basic hand functions, the reconstructed thumb has to have adequate length, proper position, large web space, and sensation of fingertip that allow to combine with other fingers to pinch and grasp. In these above elements, many authors consider proper position the main factor and determinant of outcomes [3,5]. In thumb amputation at this level, thenar muscles were lost. In our two reconstructed thumbs, only carpometacarpal joint and interphalangeal joint are functional. Therefore, the outcomes of our patients were a surprise and suggest that good functions could be achieved by exact position and good repair of long flexor and extensor tendons and sensory nerves. In the aesthetic aspect, our reconstructed thumbs have fingertip and nail like a normal thumb tip. Our patients were satisfied with the appearance of the neothumb.

The advantages of this technique include 1) simplicity, 2) lower cost and shorter reconstruction time, 3) reduction of foot morbidity compared to trans-metatarsal great toe flap. The disadvantage is shorter length of the neothumb.

## 4 Conclusion

Thumb amputation at the MC base is disabling and challenging. Accepting shorter length, reconstruction for thumb amputation at the MC base by TGT flap is simple and could be performed more easily in a single stage.

## Funding

None.

## Ethical approval

The study was approved by the research committee, 108 Military Central Hospital, Hanoi, Vietnam.

## Consent

Written informed consent was obtained from both of the patients for publication of this case report and accompanying images. A copy of the written consent is available for review by the Editor-in-Chief of this journal on request.

## Author contribution

- Nguyen Viet Tan: follow-up and post-operative management, manuscript drafting.

- Le Van Doan: follow-up and post-operative management, manuscript drafting.

- Nguyen Viet Tien: performing the operations.

## Registration of research studies

Not applicable for a case report.

## Guarantor

Nguyen Viet Tan.

## Provenance and peer review

Not commissioned, externally peer-reviewed.

## Declaration of Competing Interest

None.
